# Supplementary material for: Transcriptome Analysis Reveals Vimentin-Induced Disruption of Cell–Cell Associations Augments Breast Cancer Cell Migration
Source: Cells. 2022 Dec 13;11(24):4035. doi: 10.3390/cells11244035 (PMC9776984; doi:10.3390/cells11244035)
Supplement: Supplementary file 1 [file cells-11-04035-s001.zip › DEGs and enrichment analysis.zip!/DEGs and enrichment analysis/WT_VIMvsVector KEGG/GSEA/WT_VIM/SYSTEMIC_LUPUS_ERYTHEMATOSUS/SYSTEMIC_LUPUS_ERYTHEMATOSUS(HSA05322).html]

Details for gene set SYSTEMIC\_LUPUS\_ERYTHEMATOSUS(HSA05322)[GSEA]

|  || Dataset | fpkm.sample |
| Phenotype | sample.cls |
| Upregulated in class | WT\_VIM |
| GeneSet | SYSTEMIC\_LUPUS\_ERYTHEMATOSUS(HSA05322) |
| Enrichment Score (ES) | 0.66371346 |
| Normalized Enrichment Score (NES) | 1.1826668 |
| Nominal p-value | 0.0 |
| FDR q-value | 0.23230027 |
| FWER p-Value | 0.498 |
Table: GSEA Results Summary

  

Fig 1: Enrichment plot: SYSTEMIC\_LUPUS\_ERYTHEMATOSUS(HSA05322)      
 Profile of the Running ES Score & Positions of GeneSet Members on the Rank Ordered List

  

| PROBE | DESCRIPTION (from dataset) | GENE SYMBOL | GENE\_TITLE | RANK IN GENE LIST | RANK METRIC SCORE | RUNNING ES | CORE ENRICHMENT || 1 | ENSG00000173372 | C1QA |  |  | 96 | Infinity | -0.0016 | Yes |
| 2 | ENSG00000224389 | C4B |  |  | 210 | Infinity | -0.0035 | Yes |
| 3 | ENSG00000275663 | HIST1H4G |  |  | 211 | Infinity | -0.0034 | Yes |
| 4 | ENSG00000250966 | AC023886.2 |  |  | 251 | Infinity | -0.0041 | Yes |
| 5 | ENSG00000277720 | AL031777.2 |  |  | 473 | Infinity | -0.0078 | Yes |
| 6 | ENSG00000196866 | HIST1H2AD |  |  | 700 | Infinity | -0.0116 | Yes |
| 7 | ENSG00000224650 | IGHV3-74 |  |  | 731 | Infinity | -0.0120 | Yes |
| 8 | ENSG00000276556 | AF254982.1 |  |  | 1030 | Infinity | -0.0171 | Yes |
| 9 | ENSG00000113600 | C9 |  |  | 1212 | Infinity | -0.0201 | Yes |
| 10 | ENSG00000271626 | AC104763.2 |  |  | 1279 | Infinity | -0.0212 | Yes |
| 11 | ENSG00000275379 | HIST1H3I |  |  | 1323 | Infinity | -0.0219 | Yes |
| 12 | ENSG00000217646 | HIST1H2BPS2 |  |  | 1325 | Infinity | -0.0218 | Yes |
| 13 | ENSG00000123569 | H2BFWT |  |  | 1491 | Infinity | -0.0246 | Yes |
| 14 | ENSG00000233733 | H2AFZP6 |  |  | 1972 | Infinity | -0.0327 | Yes |
| 15 | ENSG00000231787 | AC234782.3 |  |  | 2033 | Infinity | -0.0337 | Yes |
| 16 | ENSG00000236048 | AC013470.3 |  |  | 2073 | Infinity | -0.0343 | Yes |
| 17 | ENSG00000274559 | H2BFS |  |  | 2095 | Infinity | -0.0346 | Yes |
| 18 | ENSG00000277745 | H2AFB3 |  |  | 2281 | Infinity | -0.0377 | Yes |
| 19 | ENSG00000278637 | HIST1H4A |  |  | 2402 | 32.075 | 0.0900 | Yes |
| 20 | ENSG00000234289 | H2BFS |  |  | 2483 | 12.351 | 0.1386 | Yes |
| 21 | ENSG00000188375 | H3F3C |  |  | 2522 | 10.515 | 0.1805 | Yes |
| 22 | ENSG00000275714 | HIST1H3A |  |  | 2664 | 7.221 | 0.2073 | Yes |
| 23 | ENSG00000276903 | HIST1H2AL |  |  | 2748 | 6.459 | 0.2321 | Yes |
| 24 | ENSG00000244731 | C4A |  |  | 2782 | 6.149 | 0.2564 | Yes |
| 25 | ENSG00000276368 | HIST1H2AJ |  |  | 2850 | 5.702 | 0.2783 | Yes |
| 26 | ENSG00000184260 | HIST2H2AC |  |  | 3019 | 4.796 | 0.2949 | Yes |
| 27 | ENSG00000278705 | HIST1H4B |  |  | 3033 | 4.746 | 0.3138 | Yes |
| 28 | ENSG00000218690 | HIST1H2APS4 |  |  | 3107 | 4.510 | 0.3309 | Yes |
| 29 | ENSG00000258741 | H2AFVP1 |  |  | 3163 | 4.319 | 0.3474 | Yes |
| 30 | ENSG00000274267 | HIST1H3B |  |  | 3214 | 4.207 | 0.3636 | Yes |
| 31 | ENSG00000203812 | HIST2H2AA3 |  |  | 3346 | 3.910 | 0.3772 | Yes |
| 32 | ENSG00000278463 | HIST1H2AB |  |  | 3389 | 3.798 | 0.3918 | Yes |
| 33 | ENSG00000213440 | H2AFZP1 |  |  | 3470 | 3.690 | 0.4054 | Yes |
| 34 | ENSG00000124635 | HIST1H2BJ |  |  | 3490 | 3.678 | 0.4199 | Yes |
| 35 | ENSG00000278588 | HIST1H2BI |  |  | 3722 | 3.345 | 0.4296 | Yes |
| 36 | ENSG00000185130 | HIST1H2BL |  |  | 3794 | 3.281 | 0.4416 | Yes |
| 37 | ENSG00000277775 | HIST1H3F |  |  | 3803 | 3.274 | 0.4547 | Yes |
| 38 | ENSG00000278272 | HIST1H3C |  |  | 4137 | 2.916 | 0.4609 | Yes |
| 39 | ENSG00000196890 | HIST3H2BB |  |  | 4186 | 2.870 | 0.4717 | Yes |
| 40 | ENSG00000274641 | HIST1H2BO |  |  | 4275 | 2.802 | 0.4815 | Yes |
| 41 | ENSG00000273983 | HIST1H3G |  |  | 4376 | 2.748 | 0.4909 | Yes |
| 42 | ENSG00000184270 | HIST2H2AB |  |  | 4455 | 2.679 | 0.5005 | Yes |
| 43 | ENSG00000188486 | H2AFX |  |  | 4602 | 2.614 | 0.5085 | Yes |
| 44 | ENSG00000197153 | HIST1H3J |  |  | 5245 | 2.303 | 0.5070 | Yes |
| 45 | ENSG00000125835 | SNRPB |  |  | 5251 | 2.301 | 0.5162 | Yes |
| 46 | ENSG00000197903 | HIST1H2BK |  |  | 5469 | 2.208 | 0.5214 | Yes |
| 47 | ENSG00000273703 | HIST1H2BM |  |  | 5594 | 2.159 | 0.5281 | Yes |
| 48 | ENSG00000164032 | H2AFZ |  |  | 5660 | 2.150 | 0.5357 | Yes |
| 49 | ENSG00000278828 | HIST1H3H |  |  | 5853 | 2.078 | 0.5408 | Yes |
| 50 | ENSG00000100028 | SNRPD3 |  |  | 5906 | 2.061 | 0.5483 | Yes |
| 51 | ENSG00000105968 | H2AFV |  |  | 5929 | 2.053 | 0.5562 | Yes |
| 52 | ENSG00000217862 | HIST1H4PS1 |  |  | 5966 | 2.044 | 0.5638 | Yes |
| 53 | ENSG00000230832 | AC241584.1 |  |  | 5992 | 2.035 | 0.5717 | Yes |
| 54 | ENSG00000196747 | HIST1H2AI |  |  | 6316 | 1.971 | 0.5741 | Yes |
| 55 | ENSG00000218281 | HIST1H2APS3 |  |  | 6439 | 1.936 | 0.5799 | Yes |
| 56 | ENSG00000277075 | HIST1H2AE |  |  | 6528 | 1.917 | 0.5862 | Yes |
| 57 | novel.276 | - |  |  | 6598 | 1.903 | 0.5927 | Yes |
| 58 | ENSG00000198502 | HLA-DRB5 |  |  | 7068 | 1.802 | 0.5920 | Yes |
| 59 | ENSG00000275713 | HIST1H2BH |  |  | 7082 | 1.799 | 0.5991 | Yes |
| 60 | ENSG00000181218 | HIST3H2A |  |  | 7473 | 1.720 | 0.5994 | Yes |
| 61 | ENSG00000277224 | HIST1H2BF |  |  | 7560 | 1.704 | 0.6048 | Yes |
| 62 | ENSG00000272196 | HIST2H2AA4 |  |  | 7692 | 1.679 | 0.6094 | Yes |
| 63 | ENSG00000277157 | HIST1H4D |  |  | 7790 | 1.660 | 0.6145 | Yes |
| 64 | ENSG00000180573 | HIST1H2AC |  |  | 7835 | 1.651 | 0.6204 | Yes |
| 65 | ENSG00000180596 | HIST1H2BC |  |  | 7917 | 1.638 | 0.6257 | Yes |
| 66 | ENSG00000246705 | H2AFJ |  |  | 8240 | 1.586 | 0.6266 | Yes |
| 67 | ENSG00000179101 | AL590139.1 |  |  | 8257 | 1.584 | 0.6327 | Yes |
| 68 | ENSG00000132475 | H3F3B |  |  | 8484 | 1.549 | 0.6352 | Yes |
| 69 | ENSG00000197238 | HIST1H4J |  |  | 8623 | 1.532 | 0.6390 | Yes |
| 70 | ENSG00000214141 | ACTN4P2 |  |  | 8894 | 1.494 | 0.6405 | Yes |
| 71 | ENSG00000158373 | HIST1H2BD |  |  | 8943 | 1.489 | 0.6457 | Yes |
| 72 | ENSG00000106804 | C5 |  |  | 9106 | 1.466 | 0.6489 | Yes |
| 73 | ENSG00000220323 | HIST2H2BD |  |  | 9210 | 1.451 | 0.6530 | Yes |
| 74 | ENSG00000273542 | HIST1H4K |  |  | 9271 | 1.442 | 0.6578 | Yes |
| 75 | ENSG00000270882 | HIST2H4A |  |  | 9806 | 1.382 | 0.6543 | Yes |
| 76 | ENSG00000099284 | H2AFY2 |  |  | 9973 | 1.363 | 0.6570 | Yes |
| 77 | ENSG00000178458 | H3F3AP6 |  |  | 10317 | 1.323 | 0.6565 | Yes |
| 78 | ENSG00000229391 | HLA-DRB6 |  |  | 10539 | 1.312 | 0.6581 | Yes |
| 79 | ENSG00000196787 | HIST1H2AG |  |  | 11190 | 1.248 | 0.6521 | Yes |
| 80 | ENSG00000220875 | HIST1H3PS1 |  |  | 11425 | 1.230 | 0.6531 | Yes |
| 81 | ENSG00000167088 | SNRPD1 |  |  | 12227 | 1.162 | 0.6442 | Yes |
| 82 | ENSG00000274290 | HIST1H2BE |  |  | 12269 | 1.158 | 0.6482 | Yes |
| 83 | ENSG00000233822 | HIST1H2BN |  |  | 12317 | 1.156 | 0.6521 | Yes |
| 84 | ENSG00000273802 | HIST1H2BG |  |  | 12354 | 1.152 | 0.6561 | Yes |
| 85 | ENSG00000274997 | HIST1H2AH |  |  | 12418 | 1.148 | 0.6597 | Yes |
| 86 | ENSG00000232466 | AL356133.1 |  |  | 12626 | 1.130 | 0.6607 | Yes |
| 87 | ENSG00000159403 | C1R |  |  | 13035 | 1.099 | 0.6582 | Yes |
| 88 | ENSG00000224186 | C5orf66 |  |  | 13072 | 1.097 | 0.6621 | Yes |
| 89 | ENSG00000072110 | ACTN1 |  |  | 13251 | 1.083 | 0.6634 | Yes |
| 90 | ENSG00000197061 | HIST1H4C |  |  | 13489 | 1.065 | 0.6637 | Yes |
| 91 | ENSG00000113648 | H2AFY |  |  | 14185 | 1.012 | 0.6560 | No |
| 92 | ENSG00000176919 | C8G |  |  | 14229 | 1.008 | 0.6593 | No |
| 93 | ENSG00000236534 | H3F3BP1 |  |  | 14546 | 0.996 | 0.6580 | No |
| 94 | ENSG00000183454 | GRIN2A |  |  | 14553 | 0.996 | 0.6619 | No |
| 95 | ENSG00000163041 | H3F3A |  |  | 15640 | 0.930 | 0.6472 | No |
| 96 | ENSG00000178562 | CD28 |  |  | 15650 | 0.929 | 0.6508 | No |
| 97 | ENSG00000226908 | HIST1H2BPS3 |  |  | 15686 | 0.929 | 0.6540 | No |
| 98 | ENSG00000235946 | AC234783.1 |  |  | 15716 | 0.929 | 0.6573 | No |
| 99 | ENSG00000276180 | HIST1H4I |  |  | 15985 | 0.920 | 0.6564 | No |
| 100 | ENSG00000183598 | HIST2H3D |  |  | 16796 | 0.869 | 0.6462 | No |
| 101 | ENSG00000270276 | HIST2H4B |  |  | 16854 | 0.866 | 0.6487 | No |
| 102 | ENSG00000138385 | SSB |  |  | 17624 | 0.820 | 0.6390 | No |
| 103 | ENSG00000235655 | H3F3AP4 |  |  | 17765 | 0.812 | 0.6399 | No |
| 104 | ENSG00000101812 | H2BFM |  |  | 17872 | 0.806 | 0.6413 | No |
| 105 | ENSG00000285480 | AC021097.2 |  |  | 18006 | 0.799 | 0.6423 | No |
| 106 | ENSG00000203819 | HIST2H2BC |  |  | 18321 | 0.780 | 0.6401 | No |
| 107 | ENSG00000275506 | AC010378.1 |  |  | 18942 | 0.747 | 0.6326 | No |
| 108 | ENSG00000197837 | HIST4H4 |  |  | 18954 | 0.746 | 0.6355 | No |
| 109 | ENSG00000274750 | HIST1H3E |  |  | 18968 | 0.744 | 0.6383 | No |
| 110 | ENSG00000116747 | TROVE2 |  |  | 19508 | 0.715 | 0.6320 | No |
| 111 | ENSG00000130402 | ACTN4 |  |  | 19680 | 0.706 | 0.6319 | No |
| 112 | ENSG00000179344 | HLA-DQB1 |  |  | 20131 | 0.681 | 0.6270 | No |
| 113 | ENSG00000158406 | HIST1H4H |  |  | 20388 | 0.666 | 0.6254 | No |
| 114 | ENSG00000132109 | TRIM21 |  |  | 20452 | 0.663 | 0.6270 | No |
| 115 | ENSG00000282988 | AL031777.3 |  |  | 20496 | 0.661 | 0.6289 | No |
| 116 | ENSG00000184678 | HIST2H2BE |  |  | 20500 | 0.660 | 0.6316 | No |
| 117 | ENSG00000233514 | AL356653.1 |  |  | 21003 | 0.636 | 0.6256 | No |
| 118 | ENSG00000203814 | HIST2H2BF |  |  | 21463 | 0.612 | 0.6203 | No |
| 119 | ENSG00000276966 | HIST1H4E |  |  | 22322 | 0.556 | 0.6080 | No |
| 120 | ENSG00000125730 | C3 |  |  | 22733 | 0.527 | 0.6031 | No |
| 121 | ENSG00000236852 | BX322784.1 |  |  | 23292 | 0.489 | 0.5956 | No |
| 122 | ENSG00000196735 | HLA-DQA1 |  |  | 24861 | 0.367 | 0.5705 | No |
| 123 | ENSG00000182326 | C1S |  |  | 25226 | 0.326 | 0.5656 | No |
| 124 | ENSG00000150337 | FCGR1A |  |  | 25727 | 0.279 | 0.5582 | No |
| 125 | ENSG00000143226 | FCGR2A |  |  | 26952 | 0.121 | 0.5379 | No |
| 126 | ENSG00000039537 | C6 |  |  | 28028 | 0.000 | 0.5196 | No |
| 127 | ENSG00000234612 | H2AFZP5 |  |  | 28258 | 0.000 | 0.5157 | No |
| 128 | ENSG00000167798 | C3P1 |  |  | 28442 | 0.000 | 0.5126 | No |
| 129 | ENSG00000227211 | AL035551.1 |  |  | 29011 | 0.000 | 0.5030 | No |
| 130 | ENSG00000112936 | C7 |  |  | 29196 | 0.000 | 0.4999 | No |
| 131 | ENSG00000274183 | H2AFB1 |  |  | 29704 | 0.000 | 0.4912 | No |
| 132 | ENSG00000234816 | HIST1H2APS5 |  |  | 29729 | 0.000 | 0.4908 | No |
| 133 | ENSG00000114013 | CD86 |  |  | 30046 | 0.000 | 0.4855 | No |
| 134 | ENSG00000223345 | HIST2H2BA |  |  | 30129 | 0.000 | 0.4841 | No |
| 135 | ENSG00000276410 | HIST1H2BB |  |  | 30502 | 0.000 | 0.4778 | No |
| 136 | ENSG00000274618 | HIST1H4F |  |  | 30589 | 0.000 | 0.4763 | No |
| 137 | ENSG00000240929 | HIST2H2BB |  |  | 30591 | 0.000 | 0.4763 | No |
| 138 | ENSG00000211947 | IGHV3-21 |  |  | 31175 | NaN | 0.4664 | No |
| 139 | ENSG00000231389 | HLA-DPA1 |  |  | 31322 | NaN | 0.4640 | No |
| 140 | ENSG00000146047 | HIST1H2BA |  |  | 31366 | NaN | 0.4633 | No |
| 141 | ENSG00000204287 | HLA-DRA |  |  | 31438 | NaN | 0.4621 | No |
| 142 | ENSG00000231461 | HLA-DPA2 |  |  | 31783 | NaN | 0.4563 | No |
| 143 | ENSG00000211938 | IGHV3-7 |  |  | 31870 | NaN | 0.4549 | No |
| 144 | ENSG00000211937 | IGHV2-5 |  |  | 31999 | NaN | 0.4527 | No |
| 145 | ENSG00000121594 | CD80 |  |  | 32056 | NaN | 0.4518 | No |
| 146 | ENSG00000257169 | AC125612.1 |  |  | 32129 | NaN | 0.4507 | No |
| 147 | ENSG00000278473 | IGHV3-41 |  |  | 32389 | NaN | 0.4463 | No |
| 148 | ENSG00000259990 | AC133561.3 |  |  | 32435 | NaN | 0.4456 | No |
| 149 | ENSG00000257572 | AC008127.2 |  |  | 33010 | NaN | 0.4359 | No |
| 150 | ENSG00000021852 | C8B |  |  | 33215 | NaN | 0.4324 | No |
| 151 | ENSG00000284486 | AL513315.1 |  |  | 33410 | NaN | 0.4292 | No |
| 152 | ENSG00000271507 | AC025884.3 |  |  | 33599 | NaN | 0.4260 | No |
| 153 | ENSG00000237279 | AC099677.3 |  |  | 34384 | NaN | 0.4127 | No |
| 154 | ENSG00000223931 | AC142381.1 |  |  | 34582 | NaN | 0.4094 | No |
| 155 | ENSG00000164508 | HIST1H2AA |  |  | 34618 | NaN | 0.4089 | No |
| 156 | ENSG00000226881 | H3F3AP5 |  |  | 34656 | NaN | 0.4083 | No |
| 157 | ENSG00000282639 | IGHV3-64D |  |  | 34703 | NaN | 0.4075 | No |
| 158 | ENSG00000278677 | HIST1H2AM |  |  | 34737 | NaN | 0.4070 | No |
| 159 | ENSG00000220730 | AL356057.2 |  |  | 35524 | NaN | 0.3937 | No |
| 160 | ENSG00000259505 | AC009268.1 |  |  | 35548 | NaN | 0.3934 | No |
| 161 | ENSG00000228783 | AL358976.1 |  |  | 35853 | NaN | 0.3882 | No |
| 162 | ENSG00000232216 | IGHV3-43 |  |  | 35999 | NaN | 0.3858 | No |
| 163 | ENSG00000254056 | IGHV3-71 |  |  | 36088 | NaN | 0.3844 | No |
| 164 | ENSG00000255329 | H2AFZP4 |  |  | 36418 | NaN | 0.3788 | No |
| 165 | ENSG00000254395 | IGHV4-55 |  |  | 36686 | NaN | 0.3743 | No |
| 166 | ENSG00000230618 | H3F3AP3 |  |  | 36753 | NaN | 0.3732 | No |
| 167 | ENSG00000259389 | H3F3AP1 |  |  | 36884 | NaN | 0.3711 | No |
| 168 | ENSG00000250705 | AC008526.1 |  |  | 36897 | NaN | 0.3709 | No |
| 169 | ENSG00000253173 | H2AFZP2 |  |  | 37365 | NaN | 0.3630 | No |
| 170 | ENSG00000211950 | IGHV1-24 |  |  | 37410 | NaN | 0.3623 | No |
| 171 | ENSG00000275221 | HIST1H2AK |  |  | 37587 | NaN | 0.3593 | No |
| 172 | ENSG00000259338 | AC051619.2 |  |  | 37695 | NaN | 0.3576 | No |
| 173 | ENSG00000258179 | AC079598.3 |  |  | 37730 | NaN | 0.3570 | No |
| 174 | ENSG00000270318 | IGHV3OR16-11 |  |  | 37876 | NaN | 0.3546 | No |
| 175 | ENSG00000227430 | AC019185.1 |  |  | 38085 | NaN | 0.3511 | No |
| 176 | ENSG00000255295 | AP002404.1 |  |  | 38129 | NaN | 0.3504 | No |
| 177 | ENSG00000213493 | ACTN4P1 |  |  | 38161 | NaN | 0.3499 | No |
| 178 | ENSG00000243977 | AC125604.1 |  |  | 38610 | NaN | 0.3424 | No |
| 179 | ENSG00000229674 | AL121578.2 |  |  | 39010 | NaN | 0.3356 | No |
| 180 | ENSG00000211951 | IGHV2-26 |  |  | 39086 | NaN | 0.3344 | No |
| 181 | ENSG00000211952 | IGHV4-28 |  |  | 39087 | NaN | 0.3344 | No |
| 182 | ENSG00000211955 | IGHV3-33 |  |  | 39088 | NaN | 0.3345 | No |
| 183 | ENSG00000211957 | IGHV3-35 |  |  | 39090 | NaN | 0.3345 | No |
| 184 | ENSG00000211958 | IGHV3-38 |  |  | 39091 | NaN | 0.3345 | No |
| 185 | ENSG00000211959 | IGHV4-39 |  |  | 39092 | NaN | 0.3346 | No |
| 186 | ENSG00000244429 | AC104629.1 |  |  | 39100 | NaN | 0.3345 | No |
| 187 | ENSG00000265531 | FCGR1CP |  |  | 39439 | NaN | 0.3288 | No |
| 188 | ENSG00000233627 | C4A-AS1 |  |  | 39663 | NaN | 0.3250 | No |
| 189 | ENSG00000159189 | C1QC |  |  | 39787 | NaN | 0.3230 | No |
| 190 | ENSG00000211972 | IGHV3-66 |  |  | 40289 | NaN | 0.3145 | No |
| 191 | ENSG00000231475 | IGHV4-31 |  |  | 40595 | NaN | 0.3094 | No |
| 192 | ENSG00000248993 | AL645941.2 |  |  | 40610 | NaN | 0.3092 | No |
| 193 | ENSG00000223383 | HIST1H2APS6 |  |  | 40672 | NaN | 0.3082 | No |
| 194 | ENSG00000270472 | IGHV3OR16-9 |  |  | 41081 | NaN | 0.3013 | No |
| 195 | ENSG00000234586 | AL161449.1 |  |  | 41386 | NaN | 0.2962 | No |
| 196 | ENSG00000242574 | HLA-DMB |  |  | 41446 | NaN | 0.2952 | No |
| 197 | ENSG00000237398 | HLA-DPA3 |  |  | 41625 | NaN | 0.2922 | No |
| 198 | ENSG00000251118 | AC008549.3 |  |  | 41790 | NaN | 0.2895 | No |
| 199 | ENSG00000203747 | FCGR3A |  |  | 41831 | NaN | 0.2888 | No |
| 200 | ENSG00000265541 | H3F3BP2 |  |  | 42056 | NaN | 0.2851 | No |
| 201 | ENSG00000223648 | IGHV3-64 |  |  | 42070 | NaN | 0.2849 | No |
| 202 | ENSG00000220347 | AL355297.1 |  |  | 42100 | NaN | 0.2844 | No |
| 203 | ENSG00000230228 | AL590609.2 |  |  | 42388 | NaN | 0.2796 | No |
| 204 | ENSG00000275126 | HIST1H4L |  |  | 42476 | NaN | 0.2782 | No |
| 205 | ENSG00000242387 | HIST1H2APS2 |  |  | 42835 | NaN | 0.2721 | No |
| 206 | ENSG00000258570 | AC110027.1 |  |  | 43241 | NaN | 0.2653 | No |
| 207 | ENSG00000253458 | IGHVII-15-1 |  |  | 43424 | NaN | 0.2622 | No |
| 208 | ENSG00000271178 | IGHV3OR16-13 |  |  | 43445 | NaN | 0.2619 | No |
| 209 | ENSG00000259680 | AC136428.1 |  |  | 43600 | NaN | 0.2593 | No |
| 210 | ENSG00000166278 | C2 |  |  | 43621 | NaN | 0.2590 | No |
| 211 | ENSG00000277096 | AC098590.2 |  |  | 43654 | NaN | 0.2585 | No |
| 212 | ENSG00000223865 | HLA-DPB1 |  |  | 43666 | NaN | 0.2584 | No |
| 213 | ENSG00000273112 | AL590385.2 |  |  | 43877 | NaN | 0.2549 | No |
| 214 | ENSG00000224447 | HIST1H1PS2 |  |  | 44217 | NaN | 0.2491 | No |
| 215 | ENSG00000274347 | AC127381.2 |  |  | 44482 | NaN | 0.2447 | No |
| 216 | ENSG00000218502 | H2AFZP3 |  |  | 44768 | NaN | 0.2399 | No |
| 217 | ENSG00000282600 | IGHV3-69-1 |  |  | 45189 | NaN | 0.2328 | No |
| 218 | ENSG00000237178 | AC019080.2 |  |  | 45548 | NaN | 0.2268 | No |
| 219 | ENSG00000211933 | IGHV6-1 |  |  | 46010 | NaN | 0.2190 | No |
| 220 | ENSG00000226030 | HLA-DQB3 |  |  | 46208 | NaN | 0.2157 | No |
| 221 | ENSG00000211978 | IGHV5-78 |  |  | 46505 | NaN | 0.2107 | No |
| 222 | ENSG00000211970 | IGHV4-61 |  |  | 46507 | NaN | 0.2107 | No |
| 223 | ENSG00000211976 | IGHV3-73 |  |  | 46508 | NaN | 0.2107 | No |
| 224 | ENSG00000211974 | IGHV2-70D |  |  | 46509 | NaN | 0.2108 | No |
| 225 | ENSG00000211967 | IGHV3-53 |  |  | 46749 | NaN | 0.2067 | No |
| 226 | ENSG00000102245 | CD40LG |  |  | 46826 | NaN | 0.2055 | No |
| 227 | ENSG00000253482 | IGHVII-26-2 |  |  | 47197 | NaN | 0.1993 | No |
| 228 | ENSG00000225698 | IGHV3-72 |  |  | 47239 | NaN | 0.1986 | No |
| 229 | ENSG00000237541 | HLA-DQA2 |  |  | 47428 | NaN | 0.1954 | No |
| 230 | ENSG00000100448 | CTSG |  |  | 47538 | NaN | 0.1936 | No |
| 231 | ENSG00000277858 | H2AFB2 |  |  | 47551 | NaN | 0.1935 | No |
| 232 | ENSG00000136634 | IL10 |  |  | 47625 | NaN | 0.1923 | No |
| 233 | ENSG00000162747 | FCGR3B |  |  | 47897 | NaN | 0.1877 | No |
| 234 | ENSG00000197476 | AC034105.1 |  |  | 47954 | NaN | 0.1868 | No |
| 235 | ENSG00000274576 | IGHV2-70 |  |  | 48508 | NaN | 0.1774 | No |
| 236 | ENSG00000226222 | AC079168.1 |  |  | 48929 | NaN | 0.1703 | No |
| 237 | ENSG00000168148 | HIST3H3 |  |  | 49259 | NaN | 0.1648 | No |
| 238 | ENSG00000211942 | IGHV3-13 |  |  | 49687 | NaN | 0.1576 | No |
| 239 | ENSG00000211941 | IGHV3-11 |  |  | 49688 | NaN | 0.1576 | No |
| 240 | ENSG00000211946 | IGHV3-20 |  |  | 49690 | NaN | 0.1576 | No |
| 241 | ENSG00000211944 | IGHV3-16 |  |  | 49691 | NaN | 0.1577 | No |
| 242 | ENSG00000211949 | IGHV3-23 |  |  | 49692 | NaN | 0.1577 | No |
| 243 | ENSG00000178762 | HIST1H2BPS1 |  |  | 50165 | NaN | 0.1497 | No |
| 244 | ENSG00000229421 | AC133141.1 |  |  | 50794 | NaN | 0.1391 | No |
| 245 | ENSG00000231712 | AC104698.1 |  |  | 50966 | NaN | 0.1362 | No |
| 246 | ENSG00000197409 | HIST1H3D |  |  | 51146 | NaN | 0.1332 | No |
| 247 | ENSG00000253957 | IGHV3-22 |  |  | 51989 | NaN | 0.1190 | No |
| 248 | ENSG00000197561 | ELANE |  |  | 52546 | NaN | 0.1096 | No |
| 249 | ENSG00000273079 | GRIN2B |  |  | 52664 | NaN | 0.1076 | No |
| 250 | ENSG00000216436 | HIST1H2APS1 |  |  | 52770 | NaN | 0.1059 | No |
| 251 | ENSG00000226436 | AC002367.1 |  |  | 52808 | NaN | 0.1053 | No |
| 252 | ENSG00000224557 | HLA-DPB2 |  |  | 53075 | NaN | 0.1008 | No |
| 253 | ENSG00000157131 | C8A |  |  | 53237 | NaN | 0.0981 | No |
| 254 | ENSG00000271130 | IGHV3OR16-8 |  |  | 53358 | NaN | 0.0961 | No |
| 255 | ENSG00000181201 | HIST3H2BA |  |  | 53650 | NaN | 0.0912 | No |
| 256 | ENSG00000259490 | IGHV3OR15-7 |  |  | 53830 | NaN | 0.0882 | No |
| 257 | ENSG00000278510 | AC007908.1 |  |  | 53899 | NaN | 0.0871 | No |
| 258 | ENSG00000240912 | AC092896.1 |  |  | 54003 | NaN | 0.0854 | No |
| 259 | ENSG00000270550 | IGHV3-30 |  |  | 54022 | NaN | 0.0851 | No |
| 260 | ENSG00000229776 | C4B-AS1 |  |  | 54204 | NaN | 0.0821 | No |
| 261 | ENSG00000257287 | AC016256.1 |  |  | 54266 | NaN | 0.0811 | No |
| 262 | ENSG00000277836 | AC141272.1 |  |  | 54407 | NaN | 0.0787 | No |
| 263 | ENSG00000253714 | IGHVII-53-1 |  |  | 54703 | NaN | 0.0738 | No |
| 264 | ENSG00000253883 | IGHV3-19 |  |  | 55017 | NaN | 0.0685 | No |
| 265 | ENSG00000253132 | IGHV3-62 |  |  | 55021 | NaN | 0.0685 | No |
| 266 | ENSG00000275063 | AC233755.1 |  |  | 55145 | NaN | 0.0664 | No |
| 267 | ENSG00000234202 | AC104332.2 |  |  | 55312 | NaN | 0.0636 | No |
| 268 | ENSG00000213244 | HIST2H3DP1 |  |  | 55356 | NaN | 0.0630 | No |
| 269 | ENSG00000259842 | IGHV3OR16-16 |  |  | 55539 | NaN | 0.0599 | No |
| 270 | ENSG00000259303 | IGHV2OR16-5 |  |  | 55690 | NaN | 0.0574 | No |
| 271 | ENSG00000223951 | AL133244.1 |  |  | 55714 | NaN | 0.0570 | No |
| 272 | ENSG00000255492 | AC104383.1 |  |  | 55751 | NaN | 0.0565 | No |
| 273 | ENSG00000230111 | AC115283.1 |  |  | 55765 | NaN | 0.0563 | No |
| 274 | ENSG00000204252 | HLA-DOA |  |  | 55863 | NaN | 0.0547 | No |
| 275 | ENSG00000111537 | IFNG |  |  | 56421 | NaN | 0.0453 | No |
| 276 | ENSG00000241106 | HLA-DOB |  |  | 56780 | NaN | 0.0392 | No |
| 277 | ENSG00000232810 | TNF |  |  | 56842 | NaN | 0.0382 | No |
| 278 | ENSG00000224373 | IGHV4-59 |  |  | 56993 | NaN | 0.0357 | No |
| 279 | ENSG00000211965 | IGHV3-49 |  |  | 57083 | NaN | 0.0342 | No |
| 280 | ENSG00000211964 | IGHV3-48 |  |  | 57084 | NaN | 0.0343 | No |
| 281 | ENSG00000211966 | IGHV5-51 |  |  | 57085 | NaN | 0.0343 | No |
| 282 | ENSG00000249467 | AL592156.1 |  |  | 57550 | NaN | 0.0265 | No |
| 283 | ENSG00000261834 | IGHV3OR16-15 |  |  | 58052 | NaN | 0.0180 | No |
| 284 | ENSG00000283607 | IGHVII-30-21 |  |  | 58191 | NaN | 0.0157 | No |
| 285 | ENSG00000277856 | AC233755.2 |  |  | 58276 | NaN | 0.0143 | No |
| 286 | ENSG00000173369 | C1QB |  |  | 58330 | NaN | 0.0135 | No |
| 287 | ENSG00000223497 | AC063979.1 |  |  | 58367 | NaN | 0.0129 | No |
| 288 | ENSG00000204257 | HLA-DMA |  |  | 58607 | NaN | 0.0089 | No |
| 289 | ENSG00000101017 | CD40 |  |  | 58851 | NaN | 0.0048 | No |
| 290 | ENSG00000233732 | IGHV3OR16-10 |  |  | 59090 | NaN | 0.0008 | No |
Table: GSEA details [plain text format]

  

Fig 2: SYSTEMIC\_LUPUS\_ERYTHEMATOSUS(HSA05322)      
 Blue-Pink O' Gram in the Space of the Analyzed GeneSet

  

Fig 3: SYSTEMIC\_LUPUS\_ERYTHEMATOSUS(HSA05322): Random ES distribution      
 Gene set null distribution of ES for **SYSTEMIC\_LUPUS\_ERYTHEMATOSUS(HSA05322)**
